# Capturing Early Changes in the Marine Bacterial Community as a Result of Crude Oil Pollution in a Mesocosm Experiment

**DOI:** 10.1264/jsme2.ME17082

**Published:** 2017-11-28

**Authors:** Adriana Krolicka, Catherine Boccadoro, Mari Mæland Nilsen, Thierry Baussant

**Affiliations:** 1 International Research Institute of Stavanger (IRIS), Environment department—Mekjarvik 12 4070 Randaberg Norway

**Keywords:** crude oil, microbial communities, molecular markers, environmental monitoring, rapid responders

## Abstract

The results of marine bacterial community succession from a short-term study of seawater incubations at 4°C to North Sea crude oil are presented herein. Oil was used alone (O) or in combination with a dispersant (OD). Marine bacterial communities resulting from these incubations were characterized by a fingerprinting analysis and pyrosequencing of the 16S rRNA gene with the aim of 1) revealing differences in bacterial communities between the control, O treatment, and OD treatment and 2) identifying the operational taxonomic units (OTUs) of early responders in order to define the bacterial gene markers of oil pollution for *in situ* monitoring.

After an incubation for 1 d, the distribution of the individual ribotypes of bacterial communities in control and oil-treated (O and OD) tanks differed. Differences related to the structures of bacterial communities were observed at later stages of the incubation. Among the early responders identified (*Pseudoalteromonas*, *Sulfitobacter*, *Vibrio*, *Pseudomonas*, *Glaciecola*, *Neptunomonas*, *Methylophaga*, and *Pseudofulvibacter*), genera that utilize a disintegrated biomass or hydrocarbons as well as biosurfactant producers were detected. None of these genera included obligate hydrocarbonoclastic bacteria (OHCB). After an incubation for 1 d, the abundances of *Glaciecola* and *Pseudofulvibacter* were approximately 30-fold higher in the OD and O tanks than in the control tank. OTUs assigned to the *Glaciecola* genus were represented more in the OD tank, while those of *Pseudofulvibacter* were represented more in the O tank. We also found that 2 to 3% of the structural community shift originated from the bacterial community in the oil itself, with *Polaribacter* being a dominant bacterium.

Hydrocarbon (HC)-degrading bacteria are ubiquitous in seawater. However, in pristine environments, except in areas of natural seeps of oil and natural gas, HC-degrading bacteria represent a very small fraction of the total bacterial community (3, 59). This is particularly true for obligate hydrocarbonoclastic bacteria (OHCB) (59). In contrast, in oil-polluted environments, bacteria utilizing petroleum HCs may constitute up to 100% of viable microorganisms (3). An increased abundance of bacteria that utilize short- and medium-length alkanes is typically observed first, *e.g.* representatives of *Alcanivorax*, followed by an increase in microorganisms that degrade aromatic HCs (*e.g. Cycloclasticus*) (12, 23). In the Deepwater Horizon Oil (DHO) Spill in the Gulf of Mexico, early sampling of the plume (after approximately one month) revealed the dominance of *Oceanospirallaceae*, a family including *Alcanivorax*, which formed up to 90% of the total bacterial community. The plume community was subsequently dominated by members affiliated to *Cycloclasticus* and *Colwellia* (14, 19, 24, 48). This shift in the marine bacterial community may be related to the bloom of OHCB, including species assigned to *Alcanivorax*, *Thalassolituus*, *Oleispira*, *Oleiphilus*, and *Cycloclasticus* (59) and non-OHCB such as *Marinobacter*, *Shewanella*, *Neptunomonas*, *Halomonas*, *Colwellia*, and *Shingomonas*. Regarding *Colwellia* species, which are very often associated with the early stages of petroleum pollution (6, 11, 19, 48), their blooming sometimes occurs in relation to specific and characteristic metabolic features, for example, high bicarbonate assimilation under conditions of starvation (1). Succession in marine bacterial community compositions following oil exposure is related to many factors: the weathering, degradation (*i.e.* changes in chemical composition), and bioavailability of crude oil residues, the organisms initially present in the environment, the type of oil, and external conditions such as wind, waves, and temperature (32). In cold marine environments, arctic and subarctic bacteria within *Gammaproteobacteria* are often a predominant class following exposure to crude oils (6, 10, 12, 21). Brakstad *et al.* (11) found that the *Gammaproteobacteria* species *Colwellia*, *Marinomonas*, and *Glaciecola* were predominant in contaminated ice cores in Svalbard, while clean ice included more heterogeneous populations. Gerdes *et al.* (21) also showed that other species within *Gammaproteobacteria* assigned to *Marinobacter*, *Shewanella*, and *Pseudomonas* were enriched during crude oil exposure in arctic sea-ice. Prabagaran *et al.* (44) found clones affiliated to *Psychrobacter* and others in seawater from Sub-Antarctica exposed to crude oil, but not those belonging to *Gammaproteobacteria*; *Arcobacter*, *Formosa algae*, *Polaribacter*, *Ulvibacter*, and *Tenacibaculum*.

Crude oil is inhabited by a diverse microbial community with different levels of metabolic activities (28, 36, 57). Korenblum *et al.* (28) found that the composition of indigenous bacterial communities in different crude oils varied with the water content; however, the relative abundance of *Pseudoalteromonaceae* was very high in all communities. Nevertheless, the extent to which the bacterial communities present in crude oil influence microbial composition changes, and, thus, the fate of oil in seawater after a spill currently remains unclear.

Oil bioavailability in seawater may vary markedly depending on several factors, including sea conditions or the use of additive chemicals used in spill bioremediation. Previous studies demonstrated that these chemicals exert a number of effects, from representing a toxic hazard to being useful as an additional carbon source for bacterial growth (14, 31, 33).

Beyond a detailed understanding of bacterial diversity and changes in the bacterial community following oil exposure, a major objective of the present study was to identify specific bacteria responding rapidly to the presence of oil in subarctic seawater for their use in future *in situ* monitoring technologies. The rationale for the identification and selection of these ‘rapid responders’ is anchored in the understanding that the ocean microbial community is highly sensitive to the presence of released HCs and is an effective biosensor for contamination in this environment (19). The long-term vision of this study is to integrate molecular assays recognizing specific bacterial markers of oil pollution into a genosensor device for real-time *in situ* oil detection in the context of petroleum operations and monitoring in cold regions.

A mesocosm experiment was conducted using natural seawater and a low mixing energy set-up for oil exposure. After oil addition to the surface of seawater (4°C), two scenarios were considered: one with no addition of chemicals (oil only: O), and one with a dispersant in addition to oil (oil+dispersant: OD). We used 16S rRNA gene PCR-DGGE and 454 high-throughput next generation sequencing (NGS) to characterize early bacterial community changes and identify specific bacterial genes following crude oil addition to seawater for environmental biomonitoring purposes.

## Materials and Methods

### Experimental setup

**Fig. 1:**
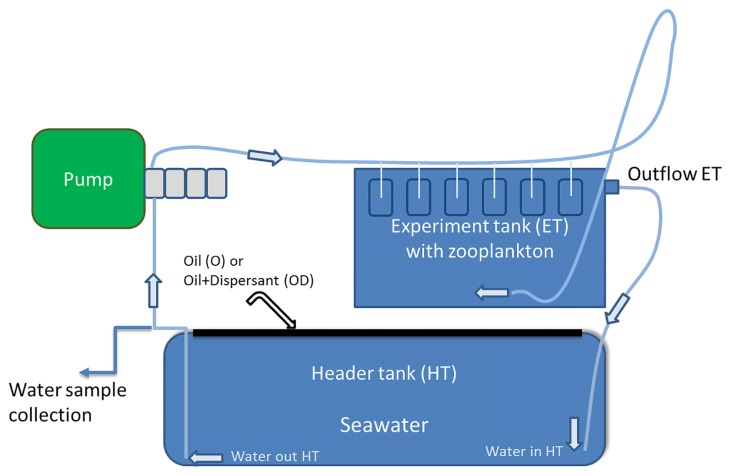
Schematic drawing of the experimental set-up with oil (O) and oil+dispersant (OD) using semi-continuous close flow seawater circulation.

A modified version of the recirculating exposure system described by Bado-Nilles *et al.* (4) was used (Fig. 1). The experiment was run at 4°C and included two treatment tanks; Oil (O) and Oil+Dispersant (OD) in addition to one Control (C) tank, each with a volume of 200 L and a surface area of 100 cm×100 cm. The C tank was kept in a separate climate room to reduce contamination. Atlantic seawater (34 PSU) was pumped from a depth of 80 m in Byfjord (59° 1′ N, 5° 37′ E) near Mekjarvik, Norway, to our research facility in which it was sand filtered. *In situ* temperature and salinity in Byfjord seawater registered with a CTD for one month during the period of the experiment were in the ranges of 4–7.3°C (mean: 6.42°C) and 32–34 PSU (mean: 32.46 PSU), respectively. At the onset of the experiment, crude oil from a field in the North Sea (North Sea oil, Table S1) was added (1 mL L^−1^) to the surface of the O tank containing seawater. In OD, 4 mL (2% oil [v/v]) of Corexit 9500A (Biologge AS, Sandefjord, Norway) was mixed with the oil, and then added to the surface of the tank. The set-up used was a low-energy system providing the gentle circulation of water without breaking the oil slick. Bottom water was pumped in a circulating loop from the C, O, and OD tanks to smaller glass tanks (10 L) with test organisms (zooplankton), used in a parallel effect study, and back to the header tank using peristaltic pumps. All intakes and outlets were placed below the surface with no contact with the oil surface layer in order to avoid direct contamination. The duration of the experiment was 28 d.

### Chemical analysis

Water samples (2 L) for characterization and the analysis of polyaromatic compounds (PAC) were collected from all tanks in Schott glass flasks 1 h, 2 d, and 7 d after the addition of O and OD. One percent hydrochloric acid was added to the samples to prevent any biodegradation prior to the GC-MS analysis (Gas Chromatograph GC-2010; Shimadzu, Tokyo, Japan) of the standard 16 polycyclic aromatic hydrocarbons (PAHs) listed by the Environmental Protection Agency (US EPA) (27). C1–C3 alkylated forms for naphthalene, phenanthrene/anthracene, and dibenzothiophene were also analyzed. Particle size distribution and the concentration of oil particles were measured with a Multisizer 3 Coulter Counter (Beckman Coulter, Fullerton, CA, USA) using an orifice tube aperture size of 100 μm with an effective analytical range of 2.0–60 μm.

### Sampling and total DNA extraction from seawater

A volume of 800–1,000 mL seawater was collected at the bottom of the tanks using a silicon hose fixed at the edge of the tanks to prevent contamination from the upper surface layer of oil at each sampling time (day 1, 2, 7, 14, and 28). Samples were immediately filtered through nitrocellulose filters (pore size, 0.22 μm) (Millipore, Billerica, MA, USA) and the filters were stored at −80°C until DNA extraction. DNA extraction was performed using the protocol described by Preston *et al.* (45) with the following modifications: the total volume of the lysate was used after filtration through a Millex-GV syringe filter (pore size, 0.2 μm; diameter, 13 mm) (Millipore) and a proportionally higher volume of diluent (555 mM sodium acetate pH 5.2 in 70% ethanol [v/v]) was added. This was then passed through a column in the DNeasy Tissue Kit (Qiagen, Hilden, Germany) and washed twice before DNA was eluted with 80 μL elution buffer.

### Total DNA isolation from North Sea crude oil

Twenty-five milliliters of the North Sea oil used in the present study was incubated for 3×7 min on ice at 100°C with 1-min intervals. The same volume (25 mL) of phenol-chloroform-isoamyl alcohol and 2 mL of distilled water was added, then mixed thoroughly by inversion and centrifugation (3,380×*g* for 6 min). The upper layer was carefully removed and discarded. The lower layer was transferred to a new tube, and an equal volume of a mixture of ethanol and sodium acetate (8:1), pH 5.2, was added. Five milliliters of the mixture was passed through a column in the DNeasy Tissue Kit (Qiagen) and processed according to the manufacturer’s instructions, with two exceptions: the second washing was performed twice and the final centrifugation was performed at 13,520×*g* for 4 min. DNA was eluted with 60 μL of elution buffer. The concentration of extracted DNA was estimated using a NanoDrop spectrophotometer (Thermo Fisher Scientific, Waltham, MA, USA).

### Denaturing Gradient Gel Electrophoresis (DGGE), amplification, and sequencing of selected bands

The primers 341F (5′-GC *clamp*-CCTACGGGAGGCAGCA-3′) (34) and SD907r (5′-CCCCGTCAATTCCTTTGAGTT-3′) (11, 41, 55) were used to amplify the bacterial V3–V4 hypervariable region of the 16S rRNA gene. The PCR mastermix contained: 2.5 units of Taq DNA Polymerase (Sigma-Aldrich, St. Louis, MO, USA) 1×buffer without MgCl_2_, 250 μg of BSA, 2 μL of the DNA template, 200 μM of each dNTP, 2.5 mM of MgCl_2_, 0.5 μM of each primer, and H_2_O to a final volume of 50 μL. PCR was performed under the following conditions: 3 min at 95°C followed by 32 cycles at 95°C for 30 s, at 54°C for 30 s, and at 72°C for 40 s, and then by a final extension at 72°C for 10 min. PCR products were verified by agarose electrophoresis. DGGE was performed in a denaturing gradient of formamide and urea, ranging between 30% and 60% (41), and run for 16 h at 80 V. A fingerprinting analysis of DGGE patterns was performed using Pearson’s correlation coefficient implemented in GelCompare II software (Applied Maths, Kortrijk, Belgium). DNA fragments from bands visible only in O or OD were extracted using elution buffer (0.5 mM ammonium acetate, 0.1% SDS, 10 mM magnesium acetate, and 1 mM EDTA) (58), re-amplified, and sequenced on an Applied Biosystems 3,730xl DNA Analyzer (Thermo Fisher Scientific). Archaeal 16S rRNA genes were not amplified from the DNA templates by PCR after repeated attempts using the following archaeal primers: ARC344f and 517r (7).

### 454 amplicon sequencing and data analysis

The 16S rRNA gene was amplified using fusion primers designed according to Roche recommendations and containing specific sequences targeted on the bacterial V3–V4 region, 5′-CTACGGGN GGCWGCAG-3′ (Bakt_340F) and 5′-GACTACHVGGGTATCTA ATCC-3′ (Bakt_784R) (26). A pyrosequencing analysis was performed on samples from the C, O, and OD tanks from day 1 and 2 (C1, C2, O1, O2, OD1, and OD2) as well as on the North Sea crude oil sample. The PCR mastermix contained the following: 2.5 units of High Fidelity Polymerase (Roche) provided 1×buffer without MgCl_2_, 250 μg of BSA, 2 μL of the DNA template, 200 μM of each dNTP, 2.5 mM of MgCl_2_, 0.25 μM of each primer, and H_2_O to a final volume of 50 μL. PCR was performed under the following conditions: at 95°C for 3 min followed by 32 cycles at 95°C for 30 s, at 55°C for 40 s, and at 72°C for 60 s, followed by a final extension at 72°C for 10 min. Agarose electrophoresis was performed to visualize PCR products. DNA fragments of the correct lengths were extracted from the gel and recovered using the MinElute Gel Extraction Kit (Qiagen). The concentration and quality of nucleic acids were estimated by a NanoDrop spectrophotometer (Thermo Fisher Scientific). All samples were pooled in equal molar amounts and sequenced using the 454 Life Sciences GS FLX System (GS FLX) and Titanium chemistry. The initial processes (removing low quality sequences with a lower average quality score than 20 and short sequences [<150 bp], splitting according to barcodes, and trimming barcoding sequences) were performed using the pipeline available on the Ribosomal Date Project II (RDP) webpage (http://rdp.cme.msu.edu) (17). Sequences were screened against chimera structures using USEARCH 6.0 (20). The selection of non-chimeric sequences from quality fasta files was performed using the online tool available on the RDP webpage. Alpha-diversity indices (Shannon and Chao1) were calculated with defined distance units of 0.03 as the cut-off level. Following this, all non-chimeric sequence reads were processed by the NGS analysis pipeline of the SILVA rRNA gene database project (SILVAngs 1.3) (47). Each sequence was aligned using the SILVA Incremental Aligner tool (SINA v1.2.10 for ARB SVN [revision 21008]) (46). The initial steps of quality control (52) were performed and identical reads were identified. Unique reads were clustered in Operational Taxonomic Units (OTUs) per sample and the reference read of each OTU was classified. The process of dereplication and a cluster analysis were performed using cd-hit-est (version 3.1.2: http://www.bioinformatics.org/cd-hit) (35). Classification was performed by a local nucleotide BLAST search against the non-redundant version of the SILVA SSU Ref dataset using blastn (version 2.2.30+) with custom settings (13). Reads without any BLAST hits or reads with weak BLAST hits remain unclassified (where the suitable function used in the pipeline of the SILVA did not exceed the value of 93). Representative sequences of the top 220 most abundant OTUs were selected and used for sequence alignment (using MEGA5 software [53]). Additionally, sequences derived from DGGE gels and from North Sea oil were included in the analysis. Phylogenetic trees were reconstructed using the maximum likelihood method implemented in the PhyML program (v3.1/3.0 aLRT) (2). The default substitution model was selected assuming an estimated proportion of invariant sites and 4 gamma-distributed rate categories to account for the rate of heterogeneity across sites. The gamma shape parameter was estimated directly from data. Reliability for internal branches was assessed using the aLRT test (SH-Like). The output tree was transferred to the online tool: Interactive Tree of Life (iTOL) (34), and visualized.

### Nucleotide sequence accession numbers

The 16S rRNA gene sequences derived from selected DGGE bands were deposited under GeneBank/ENA/DDBJ accession numbers KJ139645 to KJ139656. Sequences obtained by 454 sequencing were deposited under accession numbers ERS392422 to ERS392427 and ERS654099.

## Results

### Chemistry

The concentration of sum PAC was similar in the O and OD treatments at all sampling times (Table 1), whereas the concentration of oil particles in the size range of 2–60 μm derived from the multisizer counter was markedly higher in the OD tank than in the O tank during the incubation, with a mean particle size in the OD tank of 7.4 μm from day 2–11 after the addition of oil. In both oil treatments, at least 87% of PAC were naphthalenes (naphthalene+C1–C3 alkylated forms). However, a slightly higher percentage of higher molecular weight PACs was detectable in the OD tank than in the O tank (Table 1).

### General observations of marine bacterial community changes over time

The shift in DGGE gel bands showed that the bacterial community in all tanks changed over time (Fig. S1A). Pearson’s correlation-based clustering analysis clearly showed differences in bacterial assemblages between the early and later stages of incubation (Fig. S1B). At all sampling times, DGGE band patterns revealed quantitative and, to some extent, qualitative differences between oil-contaminated and control samples. There were also differences, albeit much less pronounced, between the O and OD tanks. The ordination following Pearson’s correlation performed by GelCompare II software provided more information and two main clusters, A and B, were separated. These clusters coincided with a temporal shift in the bacterial community triggered by oil. Cluster A included samples from day 1 and 2, while cluster B included samples from day 7 onward. Within each cluster, there was also a difference between the C and O/OD groups. While this difference was larger towards the end of the incubation experiment, there are also differences, although less pronounced (≤9% dissimilarity), between the control and oil-contaminated tanks that already appeared on days 1 and 2 within cluster A: O and OD samples clustered more tightly than C bands, as revealed by the Gel Compare analysis. Differences in O and OD banding patterns existed on day 14 and 28, but were minor. The bacterial community appeared to be gradually re-established after day 14 and bacterial communities in contaminated tanks were then relatively stable.

The extracted rRNA gene fragments corresponding to characteristic DGGE bands (bands 1–11 in Fig. S1A) were assigned to the *Alphaproteobacteria*, *Gammaproteobacteria*, and *Flavobacteria* classes (Table S2). Sequences from characteristic bands derived from day 1 and 2 were assigned to *Shingomonas*, *Pseudoalteromonas*, *Sulfitobacter*, *Colwellia*, and *Marinomonas* genera, all previously associated with oil spills (11, 23, 48, 54). The closest relatives to the samples exposed for 14 and 28 d to oil (Fig. S1B) were genera typically associated with HC degradation; *Reinekea* sp. (band no. 8), uncultured Flavobacteria (band no. 9), uncultured *Roseobacter* (band no. 10), and the very typical psychrophilic OHCB *Oleispira* sp. gap-e-97 (band no. 11).

### Taxonomical identification of enriched bacteria

After removing chimeric sequences, which constituted 20.4%–32.7%, a similar set of sequences was included in the next analysis: day 1—40078 (C1), 35997 (O1), and 32265 (OD1) and day 2—7459 (C2), 9675 (O2), and 10294 (OD2) (Table S1. Supp.). The rarefaction analysis is presented in the supplementary material (Fig. S2). Alpha diversity expressed by the Shannon index (H′) for the control samples was 5.9 after day 1 and 5.1 after day 2 (cut-off distance unit, 0.03). In oil-polluted seawater samples, higher values for the Shannon index were obtained for both days: 6.4 (O1), 6.6 (OD1), 5.6 (O2), and 5.5 (OD2) (Table S3).

**Fig. 2:**
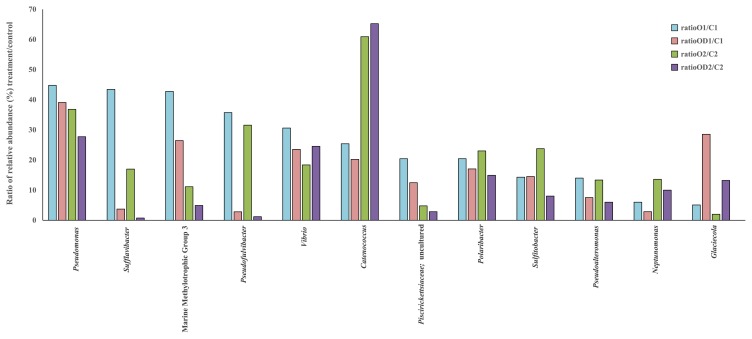
Relative abundance of the most abundant OTUs (*Colwellia* OTUs excluded) expressed as a % of each taxon (rapid responders) in O and OD treatments divided by those in the control.

**Fig. 3:**
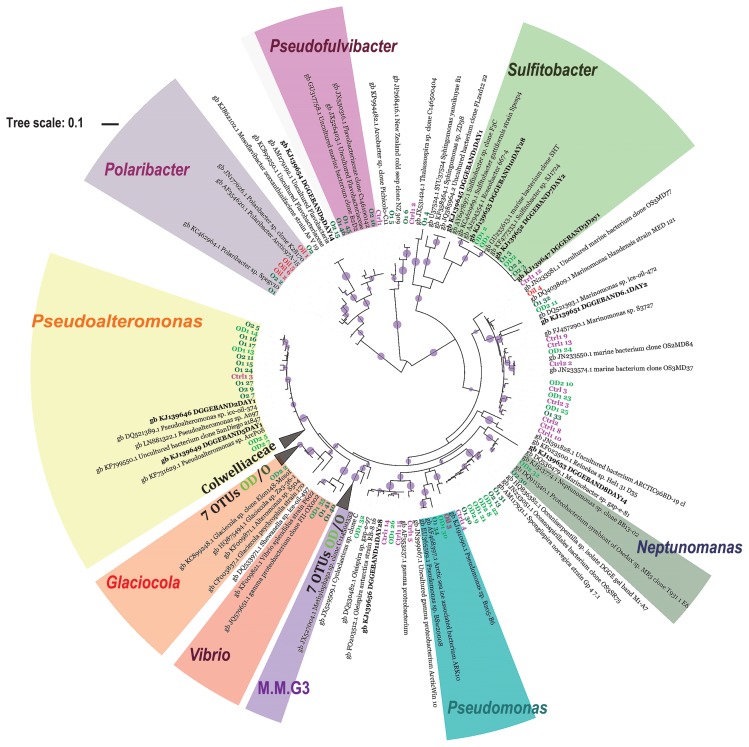
Phylogenetic tree reconstructed using the maximum likelihood method implemented in the PhyML program (v3.1/3.0 aLRT). The phylogenetic tree was based on the partial 16S rRNA gene sequence (~411 bp) of NGS top OTUs (exclusive of those assigned to the *Colwellia* genus) and sequences derived from unique DGGE gel bands. The names of OTUs derived from the C1 and C2 samples are marked in pink, and the names of OTUs from oil-polluted samples (O1, O2, OD2, OD2) are marked in dark and light green, respectively. Bold names in black are OTUs derived from DGGE. Red names correspond to OTUs derived from crude oil. The colored names of bacterial groups around the circle correspond to possible “rapid responders”, M.M.G3-Marine Methylotrophic Group 3. Bootstraps are shown for values higher than 0.8 (filled, violet circles).

Bacterial communities in the seawater samples were dominated by bacteria assigned to the class *Gammaproteobacteria*. Among these, the *Colwellia* and *Marinomonas* genera were the most abundant, constituting more than 70% and approximately 10%, respectively, in the C samples of day 1 and day 2 samples. *Colwelliaceae* with *Colwellia* was the most abundant and diverse ribotype in all samples. *Colwellia* was represented in total by 2,443, 2,093, and 2,033 OTUs in the C1, O1, and OD1 seawater samples, respectively. The analysis of the most abundant OTUs did not demonstrate a clear separation of *Colwellia* OTUs for the C and O/OD samples (Fig. S3). The bacterial community shifted in oil-contaminated samples after an incubation for 1 d. The contribution of *Colwellia* was 20% lower in the O1 and OD1 seawater samples than in the controls. While the relative contribution of *Colwellia* decreased in O and OD samples, *Pseudoalteromonas* increased (≥8-fold) in relative abundance in both treatments after 1 d, contributing to 14% of the total community in the O sample (Fig. 2). The most abundant OTUs representing the *Pseudoalteromonas* genus demonstrated a high percentage of 16S rRNA gene sequence identity to *Pseudoalteromonas* sp. ice-oil-374, the same species identified by DGGE (Fig. 3). Almost all of the most abundant *Pseudoalteromonas* OTUs were derived from O/OD seawater samples and only one was derived from the C sample. The *Glaciecola* genus (the closest relative *Glaciecola* sp. Za3-36-1 and described Uncultured *Glaciecola* sp. clone F2C28 derived from Antarctica) was more characteristic for the OD1 and OD2 samples than for the O1, O2, and C samples, achieving a relative abundance of 7 and 10% after being incubated for 1 and 2 d, respectively. In the O1 and O2 samples *Pseudofulvibacter* was more characteristic than in the OD and C samples, achieving an approximately 30-fold higher abundance (Fig. 2). The abundance of other bacteria such as *Pseudomonas*, *Vibrio*, *Piscirickettsiaceae* (Marine Methylotrophic Group 3 and uncultured *Piscirickettsiaceae*), *Polaribacter*, *Sulfitobacter*, and *Neptunomonas* was several-fold higher in O and OD samples than in C samples (Fig. 2). The most abundant OTUs of these highly-enriched bacteria were related to *Pseudomonas* sp. BSw2000, *Vibrio splendidus* strain P602, Uncultured bacterium clone; deep-sea methane seep clone, *Polaribacter* sp. Spegv10, *Sulfitobacter* sp. SJ1724, and *Sulfitobacter guttiformis*, Uncultured *Neptunomonas* sp. clone BB13-02 (Fig. 3).

### Bacterial OTUs common in North Sea oil and oil-contaminated seawater

The number of sequences in North Sea oil after both initial processes in the pipeline and chimeric sequence removal was very low at approximately 140. The reason for this may be that in the DNA extract, which was subjected to sequencing, some compounds remained that may have influenced the sequencing process. A previous study demonstrated that phenolic substances may degrade DNA polymerases (51) and PAHs may directly damage the DNA template through the formation of adducts (40, 43). Follow-up studies to overcome these difficulties are needed in order to confirm the entire microbial community of North Sea crude oil. Despite the limited number of sequencing reads from North Sea oil, 40% of the taxa were assigned to the genus *Polaribacter* (Fig. S4). The relative abundance of dominant bacteria in O and OD (*e.g. Marinomonas*, *Pseudomonas*, *Colwellia*, *Sufflavibacter*, *Glaciecola*, and *Sulfitobacter*) was in the range of 0.7% to 10.6% and those listed (Fig. S4) were common in oil-polluted samples (both O and OD). Besides the phyla *Proteobacteria* and *Bacteroidetes*, few members of *Staphylococcus* (2.8%) and *Streptococcus* (1.4%) of the phylum *Firmicutes* were detected in North Sea oil. The sequences of the 16S rRNA genes of *Marinomonas* and *Polaribacter* present in North Sea oil (labeled as Oil 4) were 99% and 98% identical, respectively, to those present in the O and OD samples (Fig. 3).

## Discussion

### General observations

The North Sea crude oil used in the present study had a PAC concentration of 1.2% (sum NPD, Table S1), a value typical of crude oil from this region (43). Based on PAC concentrations measured from GC-MS and oil particle concentrations estimated from the Multisizer (Table 1), only a small fraction (<1%) of the oil originally added at the start of the experiment (1 mL L^−1^) was present in the water phase underneath the oil slick and the dissolution of oil compounds in the water was low. This results from the low-mixing energy system used in the experimental set-up. The dispersant was used to increase the breakup of the oil layer in the OD tank (11). When a dispersant is applied to an oil slick, its effectiveness at dispersing the spilled oil depends on factors such as oil properties, wave-mixing energy, temperature, and the salinity of water (15). The effectiveness of the dispersant appeared to be low because there was not sufficient mixing energy. Since the concentration of oil particles measured in O was less than that in OD, particles larger than 60 μm may have been present in the water phase of the O tank, but were not detected by the Multisizer with an aperture size of 100 μm and a maximum effective particle detection of 60 μm, while the effect of the dispersant was to reduce the mean size of the oil particles in OD.

The oil concentration in mesocosm water was not markedly different following the treatment with or without the dispersant; therefore, no significant difference was observed in the taxonomical structure of the bacterial community between the two treatments. The small oil particles in the OD treatment may have increased the bioavailability of the oil components to the bacteria, resulting in the faster degradation of smaller PACs in OD. This may explain some of the minor microbial differences observed in O and OD.

### Detailed microbial taxonomic description

Our results were influenced by the constraint of the mesocosm and experimental set-up, the conditions of which differ to the actual field. The changes observed in the bacterial community over time in all tanks (contaminated and non-contaminated) were expected because the systems are closed and the laboratory environment will inevitably differ from *in situ* conditions. Moreover, the availability of nutrients will decline over time under all conditions. Most previous experimental studies that focused on marine bacterial microbial succession caused by compounds at low temperatures in oil were performed from the perspective of microbes actively involved in HC biodegradation (6, 8, 9, 29). The main focus was to capture the rapid oil microbial responders that significantly increase within one to two d after oil addition to the tanks, mimicking an oil spill. We observed that none of these belonged to genera that include OHCB. This means that the response from the bacterial community in the early stages comes from generalists in HC degradation, as well as from those that may utilize released organic matter in response to the presence of toxic oil components. After being incubated for 14 d, the structural changes observed in the bacterial community were more typical of HC degradation assemblages, and potentially the OHCB *Oleispira* sp..

The *Colwellia* genus has previously been linked to crude oil pollution and degradation, particularly in cold marine environments (5, 12, 39). Although chemical analyses of C seawater revealed no sign of oil contamination, this and previous studies showed the prominent contribution of *Colwellia* (previously 33%) to the composition of the natural bacterial community of seawater samples from the same seawater (Byfjord) (6). The quantification analysis on the 16S rRNA gene copies of *Colwellia* by real-time PCR, using protocols in Krolicka *et al.* (30), demonstrated that the contribution of *Colwellia* was variable and in the range of 5–50% of the total 16S rRNA bacterial gene copies in Byfjord seawater at a depth of –80 m (unpublished data, Fig. S5). The total number of bacterial 16S rRNA gene copies enumerated according to Nadkarni *et al.* (42) was low and ranged from 1.62×10^4^ to 1.09×10^5^ mL^−1^ (unpublished data, Fig. S5). The relatively high contribution of *Colwellia* in tank C may reflect the variable contribution of this genus in Byfjord seawater, possibly due to a variation in the carbon load of Byfjord seawater. Neither previous findings nor the present results demonstrated specific clusters of *Colwellia* (by using SSU rRNA) exclusively related to HCs degrading *Colwellia* (37). Follow-up studies are warranted that will allow clusters of *Colwellia* to be distinguished based on their ability to utilize HCs.

The *Pseudoalteromonas* genus was identified as an enriched genus by NGS within one d of oil exposure. These organisms may have contributed to the higher bioavailability of the oil components through the production of extracellular polymeric substances (EPS) that have the potential to serve as a biosurfactant (16), which may, in part, explain the limited differences observed in the chemistry of the O and OD tanks. A previous study demonstrated that some *Pseudoalteromonas* strains are capable of the very effective production of EPS in as short a period as one day (49). In a study on bacterial communities during an experimental oil spill in the North Sea by Chronopoulou *et al.* (16), strains assigned to *Pseudoalteromonas* constituted the majority of those isolated and were responsible for the utilization of PAHs, and branched and straight-chain alkanes in the early stages of oil contamination. These findings are consistent with the present results obtained by NGS and imply that the *Pseudoalteromonas* genus plays a significant role in the early stages of contamination. *Pseudomonas* strains have been reported to enhance the degradation of PAHs through the production of biosurfactants, which increase dispersion and emulsification (38, 44). Besides *Pseudoalteromonas* and *Pseudomonas*, isolates of *Glaciecola* from petroleum-contaminated sites along the Norwegian coastline were recently characterized as species producing biosurfactants, thereby reducing surface tension and stimulating emulsification (18). In the present study, these three genera were more abundant in oil-contaminated seawater samples, possibly explaining the limited differences observed in the chemistry of the O and OD tanks. The *Glaciecola* genus was more abundant in the OD tank than in the O tank. To the best of our knowledge, there are no data to show that the *Glaciecola* genus utilizes dispersants as an additional carbon source. The *Piscirickettsiaceae* family (MMG3 and uncultured *Piscirickettsiaceae*) (Fig. 3) increased in number in the oil-polluted experimental treatments. The closest relatively enriched genus within MMG3 *Piscirickettsiaceae* was *Methylophaga* sp. (Fig. 2). *Methylophaga* have been reported as bacteria responsible for the consumption of methane and may utilize high-molecular-weight HCs (12, 19, 24, 56). *Neptunomonas* was enriched several-fold in oil-polluted samples. An isolate from oil-contained sediments, *Neptunomonas naphthovorans*, was described as a bacterium capable of utilizing dimethylnaphthalene and phenanthrene (25) present in the oil in the present study.

The relative abundance of *Sulfitobacter* identified by NGS was higher in the O and OD treatments, particularly in O samples, than in the C treatment. The *Rhodobacteraceae* family with the *Sulfitobacter* genus was identified as the most abundant in petroleum-exposed seawater from the North Sea (9). Bælum and co-workers (5) reported that oil and oil with a dispersant stimulates bacterial cells to aggregate and form flocs (marine snow) with a high relative abundance of *Rhodobacteraceae*. In another experimental study, members of unclassified *Rhodobacteraceae* associated with algal bloom were reported to have strongly contributed to the utilization of naphthalene, to the point of complete disappearance within seven days (22). Furthermore, Størdal and co-workers (52) observed that the concentrations of viable oil-degrading *Rhodobacteraceae* family bacteria (inter alia *Sulfitobacter*) increased in feces from *Calanus finmarchicus* feeding on petroleum oil dispersions. Our study was performed in parallel to a study examining the effects of the O and OD treatments on zooplankton (krill *Meganyctiphanes norvegica* and copepods *Calanus* spp (Ingvarsdottir *et al.*, 2014, IRIS Report no. 2014/123). Oil components in the O and OD treatments may have enhanced organic waste from zooplankton and indirectly influenced microbial structural changes. The present study indicated that bacteria clades that respond to oil in the presence of a realistic ‘carbon pool’ may differ to those enhanced in an exposure experiment with a low water organic load. An experimental set-up with a realistic and seasonal carbon load is recommended for future studies investigating bacterial community changes and biodegradation processes.

The high enrichment of *Pseudofulvibacter* was previously associated with polysaccharide degradation (60), but this was not reported for oil component degradation, indicated in this study. Chitin is a biopolymer of the amino sugar glucosamine that constitutes the exoskeletons of zooplankton and other marine organisms. Hence, the abundance of *Pseudofulvibacter* may be related to a higher content of hydrolyzed or disintegrating chitinous zooplankton carapaces in oil-exposed seawater in which the mortality of zooplankton was high (Ingvarsdottir *et al.*, 2014, IRIS Report no. 2014/123). More biochemical data are needed in order to support this observation in future studies.

## Conclusions

Experimental incubations at a low temperature for one to two days were sufficient to observe a quantitative difference and also make qualitative observations of differences in the bacterial community in control and oil-contaminated seawater. Significant differences were not observed between oil alone and oil with a dispersant as a result of our experimental set-up. DGGE fingerprinting showed that structural differences in the bacterial community after one week were more pronounced. In the early stages after oil addition, blooming bacteria were not only associated with the degradation of oil components, but also those associated with a higher content of organic matter from the disintegrated biomass. The enriched bacterial OTUs identified in the early stages of oil contamination will contribute to revealing microbial gene markers of interest for oil monitoring and how fast they may be detected. These include 1) bacteria known as biosurfactant producers and HC degraders such as *Pseudoalteromonas*, *Glaciecola*, and *Pseudomonas*, 2) bacteria that may be related to a higher organic matter content such as *Pseudofulvibacter* and *Sulfitobacter*, and 3) bacteria that are often linked with the utilization of HCs such as marine methylotrophic Group 3 of *Piscirickettsiaceae*, *Neptunomonas* and *Vibrio*.

Our results suggest that some bacterial genera present in the oil itself became abundant in seawater following oil contamination, indicating that a probable origin of these bacteria is the oil itself. The impact of the bacterial community present in oil on bacterial assemblages in seawater following a pollution event needs more focus in future studies. One approach to demonstrate the fate of microorganisms in seawater originating from crude oil and their potential contribution to the utilization of oil components may be a mesocosm study with sterilized seawater as a control. It may be desirable to enumerate the phylotypes of bacteria that inhabit crude oil itself and then in oil-contaminated seawater.

Although shifts in bacterial communities were the primary focus of previous studies, our results indicate that the proper selection of target organisms needs to favor those with a ubiquitous presence in the ocean and not those specific to the crude oil microbial ‘pool’. As the vision of this project is to use bacterial gene targets as biosensors for oil detection, this selection needs to focus on organisms broadly distributed across different geographic regions. This is not a trivial task and may require more regional adaptation, particularly with regards to extreme environments *e.g.* the cold regions of the northern latitudes.

The next step in this work is to develop and validate quantitative assays for real-time *in situ* detection in seawater. Research has been initiated to quantify the abundance of a selection of oil-degrading bacteria using analytical protocols mimicking those of the Environmental Sampling Processor (30), a genosensor capable of real-time microbial gene detection and quantification at sea compatible with q-PCR chemistry (50).

## Supplementary Material



## Figures and Tables

**Table 1:** Polyaromatic hydrocarbon compounds (PAC; μg L^−1^) measured by GC-MS and estimated oil concentrations (mg L^−1^) and particle sizes (effective range, 2–60 μm) measured by a Multisizer Coulter counter in water samples collected below the surface oil slick in O and OD tanks from day 1 to 11 of the incubation. Naphthalenes: C0/C1/C2/C3-Naphthalenes; 2–3 ring PAC: Acenaphthylene, Acenaphthene, Fluorene, C0/C1/C2-Phenanthrenes, C0/C1/C2-Dibenzothiophenes; 4–6 ring PAC: Fluoranthene, Pyrene, Benzo(a)anthracene, C0/C1/C2-Chrysene, Benzo(b,j,k)fluoranthene, Benzo(a)pyrene, Indeno(1,2,3-cd)pyrene, Benzo(g,h,i)perylene, Dibenzo(a,h)anthracene.

Treatment/Sampling time		μg L^−1^ PAC (% of ∑PAC)			∑PAC μg L^−1^	Particles (2–60 μm) Mean (SD)	
	
		Naphthalenes	2–3 ring PAC	4–6 ring PAC		Conc. (mg L^−1^)	Size (μm)
Control	1 h	not detected	not detected	not detected	not detected	—	—
	2 d	0.007	not detected	not detected	0.007	—	—
	7 d	0.009	not detected	not detected	0.009	—	—
	11 d	—	—	—	—	0.7 (0.2)	14.9 (3.1)

Oil (O)	1 h	0.290 (98%)	0.007 (2%)	not detected	0.297	—	—
	2 d	4.900 (98%)	0.122 (2%)	not detected	5.022	—	—
	3 d	—	—	—	—	0.1 (0.01)	11.2 (1.6)
	7 d	2.890 (95%)	0.139 (5%)	not detected	3.029	—	—
	8 d	—	—	—	—	—	—
	9 d	—	—	—	—	0.8 (0.1)	7.6 (1.7)
	11 d	—	—	—	—	2.4 (0.1)	8.6 (0.9)

Oil+Dispersant (OD)	1 h	0.067 (100%)	not detected	not detected	0.067	—	—
	2 d	5.620 (86.7%)	0.843 (13.0%)	0.022 (0.3%)	6.485	10.8 (0.2)	9.8 (0.5)
	3 d	—	—	—	—	7.6 (0.1)	7.5 (0.3)
	4 d	—	—	—	—	6.0 (0.1)	7.0 (0.4)
	7 d	1.940 (90%)	0.190 (8.8%)	0.025 (1.2%)	2.156	—	—
	8 d	—	—	—	—	2.0 (0.1)	6.1 (0.5)
	9 d	—	—	—	—	1.8 (0.1)	6.8 (0.7)
	11 d	—	—	—	—	2.0 (0.1)	7.4 (0.7)
