# Supplementary material for: Capturing Early Changes in the Marine Bacterial Community as a Result of Crude Oil Pollution in a Mesocosm Experiment
Source: Microbes Environ. 2017 Nov 28;32(4):358–66. doi: 10.1264/jsme2.ME17082 (PMC5745021; doi:10.1264/jsme2.ME17082)
Supplement: Supplementary file 1 [file 32_358_s1.pdf]

Supplementary figure S1A, Krolicka

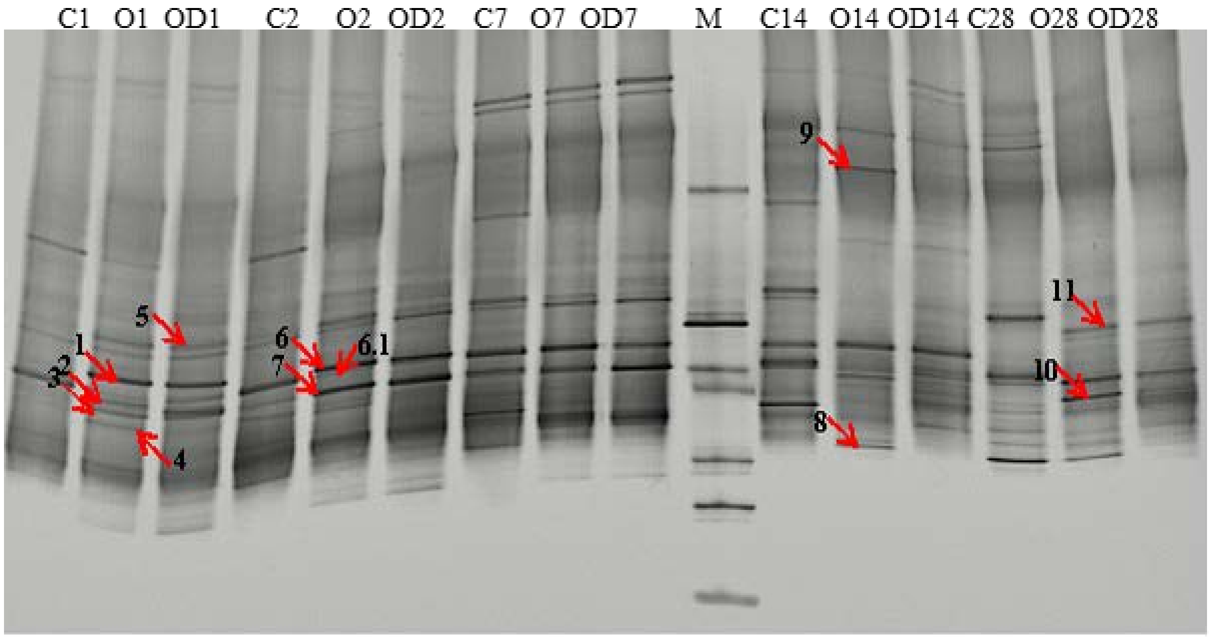

Supplementary figure S1B, Krolicka

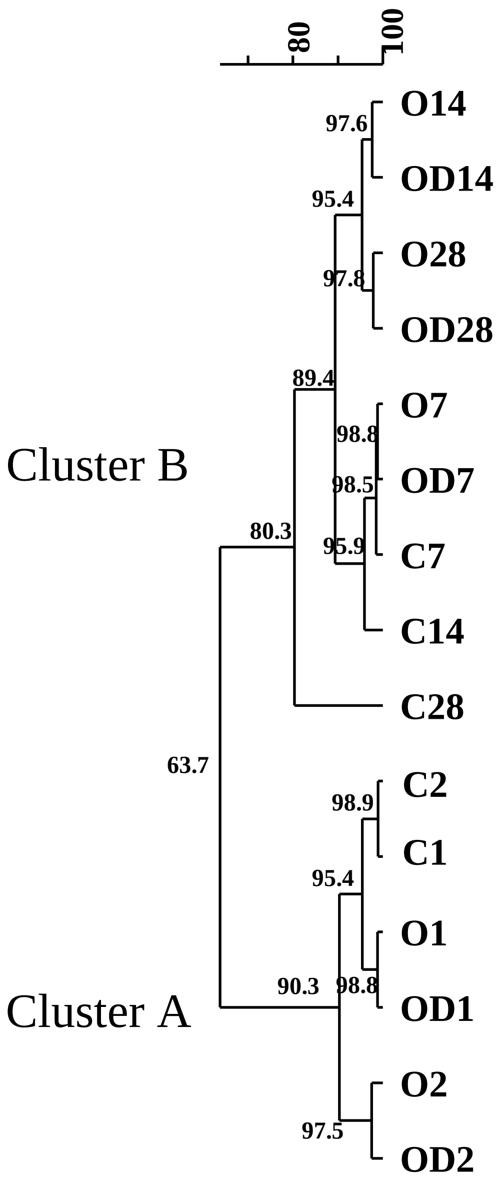

Supplementary figure S2, Krolicka

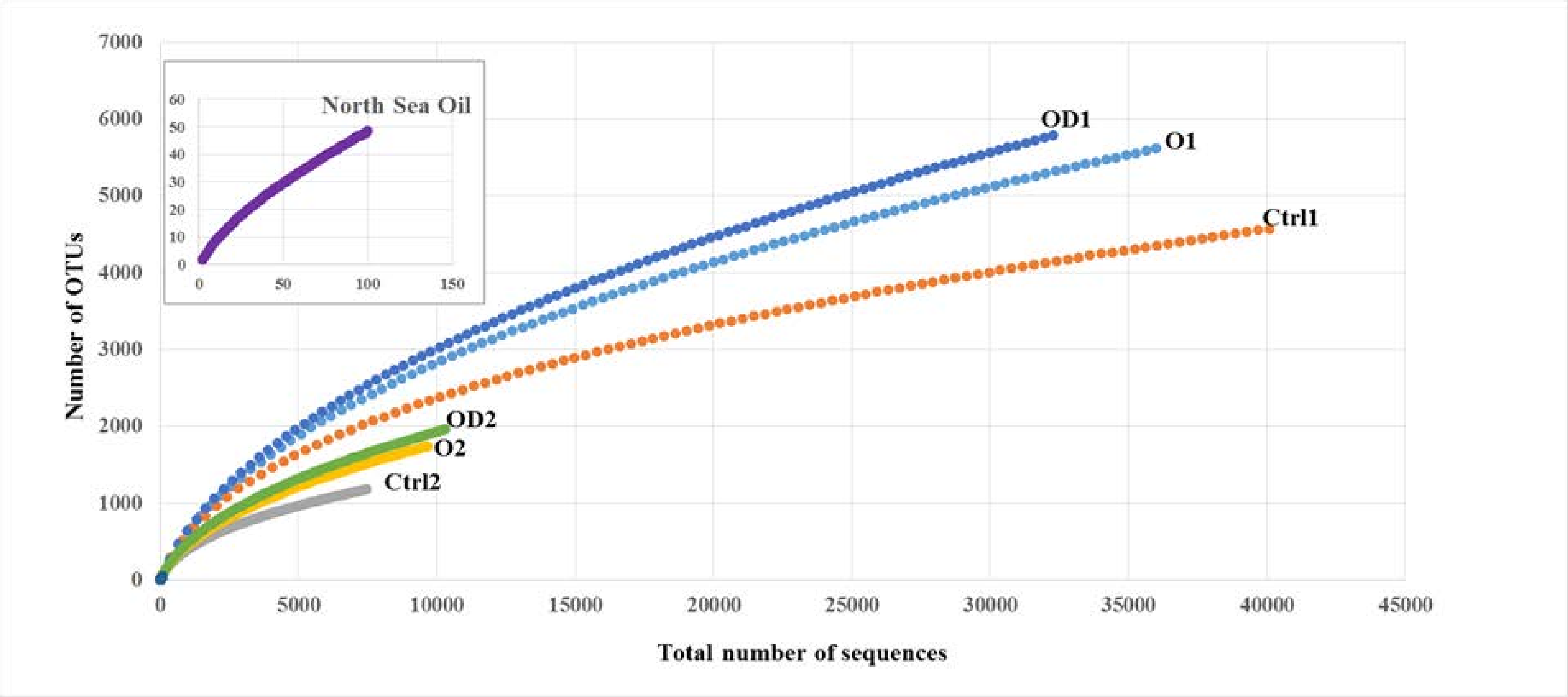

Supplementary figure S3, Krolicka

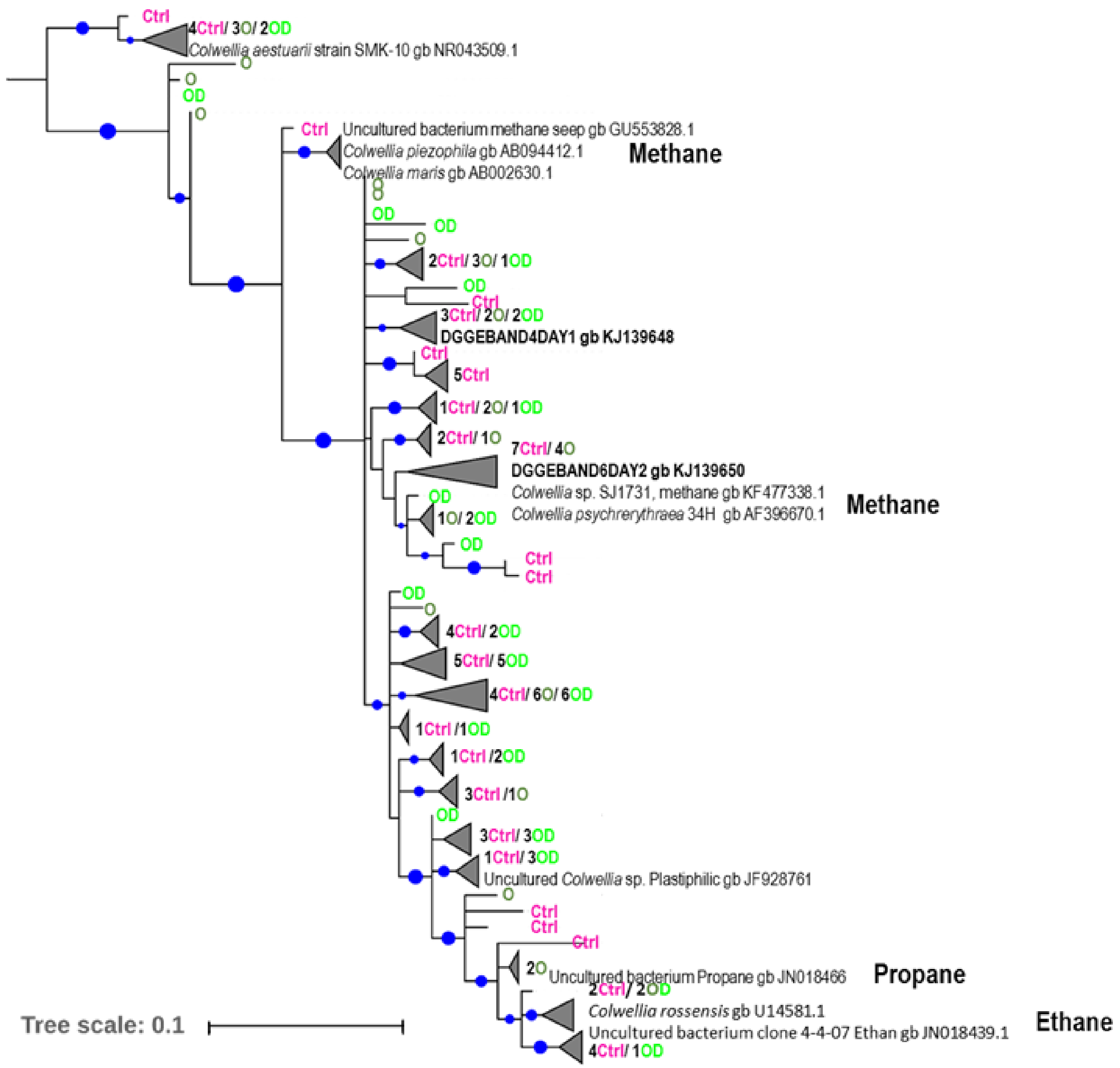

Supplementary figure S4, Krolicka

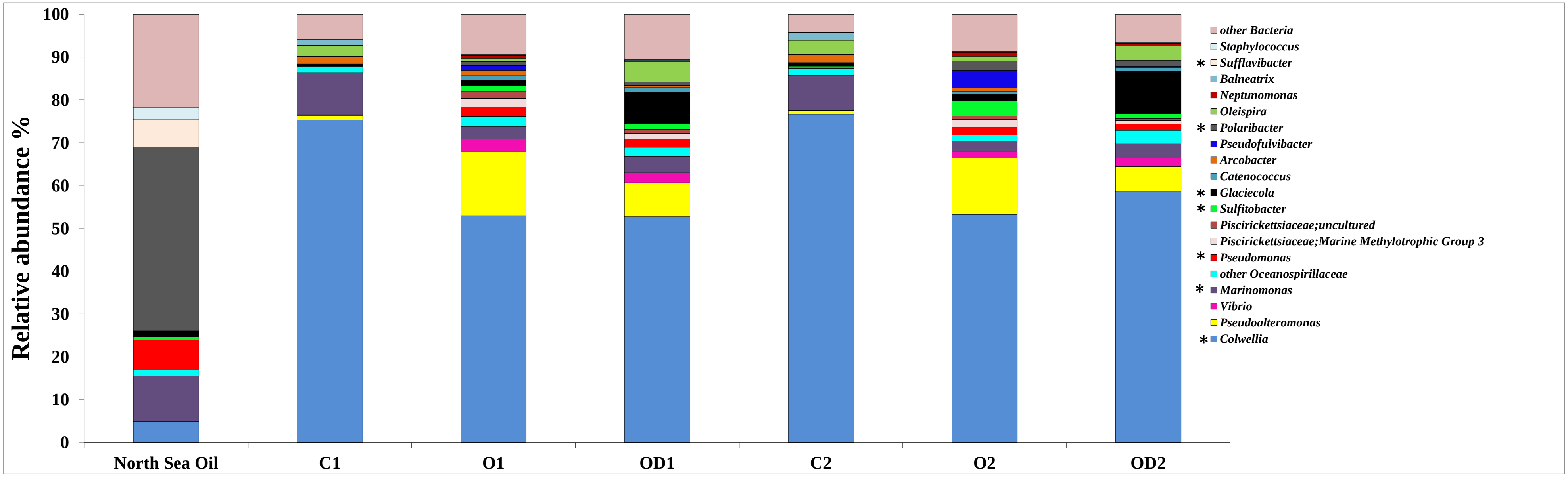

Supplementary figure S5, Krolicka

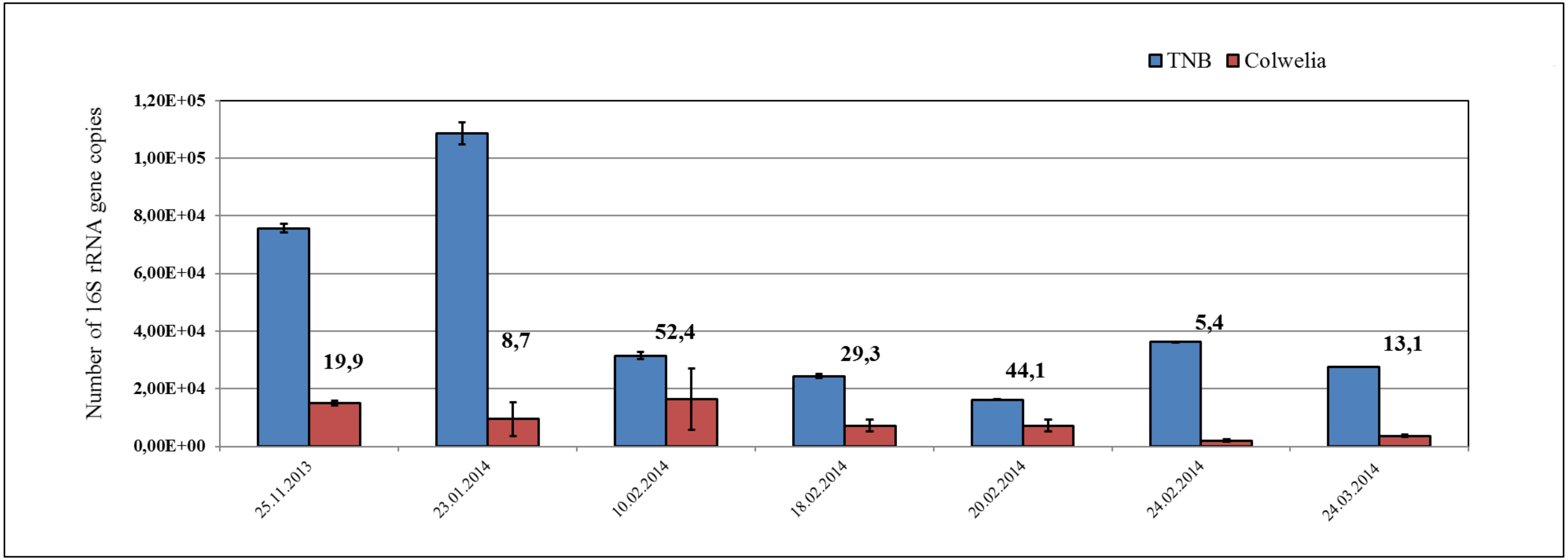

Supplementary table S1

Compound	Concentration (mg/L)
Naphthalene	880
* Sum C1- Naphthalene	2600
* Sum C2- Naphthalene	3000
* Sum C3-Naphthalene	3700
Acenaphthylene	5.7
Acenaphthene	18
Fluorene	72
Phenanthrene	280
Anthracene	1.8
* Sum C1- Phenanthrene/Anthracene	580
* Sum C2- Phenanthrene/Anthracene	830
* Sum C3- Phenanthrene/Anthracene	200
* Dibenzothiophene	32
* Sum C1- Dibenzothiophene	86
* Sum C2- Dibenzothiophene	150
* Sum C3- Dibenzothiophene	3.1
Fluoranthene	9.9
Pyrene	13
Benzo(a) anthracene	8.1
Chrysene	22
Benzo(b)fluoranthene	7.2
Benzo(k)fluoranthene	0.91
Indeno(1,2,3-c,d)pyrene	1.3
Benzo(g,h,i)perylene	3.3
Benzo(a)pyrene	3.1
Dibenz(a, h)anthracene	2.2
* Sum 16 EPA-PAH	1300
* Sum NPD	12000

Number of band	Accession number	Match in Blast analysis (accession number)	Identity scores (% similarity)	E value
1	KJ139645	*Shingomonas* sp. ZD38 (KF358364.1)	99	0.0
2	KJ139646	*Pseudoalteromonas* sp. ice-oil-374 (DQ521389.1)	98	0.0
3	KJ139647	*Sulfitobacter* sp. SJ1724 (KF477333)	99	0.0
4	KJ139648	*Colwellia* sp. SJ1731 (KF477338.1)	97	0.0
5	KJ139649	*Pseudoalteromonas* sp. ice-oil-374 (DQ521389.1)	97	0.0
6	KJ139650	*Colwellia* sp. SJ1731 (KF477338.1)	99	0.0
6.1	KJ139651	*Marinomonas* sp. S3727 (FJ457290)	95	0.0
7	KJ139652	*Sulfitobacter* sp. SJ1724 (KF477333)	99	0.0
8	KJ139653	*Reinekea* sp. (KF023500.1)	99	0.0
9	KJ139654	uncultured *Flavobacteria* (KC899250.1)	99	0.0
10	KJ139655	uncultured *Roseobacter* 667-4 (AJ294354.1)	99	0.0
11	KJ139656	*Oleispira* sp. gap-e-97 (DQ530482)	98	0.0

sample	read number	OTUs numbers	Chao1 index	Shannon index (H´)
**C1**	40078	4500	4781	5.9
**O1**	35997	5500	6762	6.4
**OD1**	32265	5800	7818	6.6
**C2**	7459	1200	1248	5.1
**O2**	9675	1600	2801	5.6
**OD2**	10294	2000	2483	5.5

Supplementary

Suppl. Figure S1A. DGGE profiles of PCR amplified bacterial 16S rRNA gene; C–control sample, O–crude oil, OD – crude oil and dispersant, numbers correspond to the day of sampling. Arrows indicate the sequenced unique DGGE bands (no 1-11).

Suppl. Figure S1B. Cluster analysis of bacterial communities (using Pearson correlation coefficient as similarity measure) based on the 16S rRNA gene PCR – DGGE profiles; C–control sample, O–crude oil, OD – crude oil and dispersant, numbers correspond to the day of sampling. The scale bar represents values of similarity.

Suppl. Figure S2. Rarefaction analysis; number of OTUs and numbers of sequences in analyzed samples

Suppl. Figure S3. Phylogenetic tree reconstructed using the maximum likelihood method implemented in the PhyML program (v3.1/3.0 aLRT). The phylogenetic tree was based on partial 16S rRNA gene sequence (~411 bp) of NGS top *Colwellia* OTUs and *Colwellia* sequences derived from DGGE gel bands responding to oil polluted samples. Names of OTUs derived from the C1 and C2 samples are marked in pink, names of OTUs from the oil polluted samples (O1, O2, OD2, OD2) are marked in dark and light green respectively. Bolded names in black are those OTUs derived from DGGE. Bootstraps are displayed for values higher than 0.8 (filled, blue circles). In order to improve readability clusters whose average branch length distance was below 0.05 were collapsed. Clusters with species known to use methane, propane and ethane are indicated .

Suppl. Figure S4. Relative abundance (%) of bacteria in the North Sea crude oil and oil contaminated seawater samples C1, O1, OD1, C2, O2, OD2 in bacterial community, asterisks by the names are common genera in the North Sea crude oil and oil contaminated seawater samples

Suppl. Figure S5. Total number of bacterial 16S rRNA gene copies (TNB) per 1 ml of Byfjord seawater (-80 m depth) and number of 16S rRNA gene copies of *Colwellia* per 1ml for different timepoints. The values above the columns respond to the contribution of 16S rRNA gene copies of *Colwellia* in TNB number (%).

Suppl. Table S1. Chemical PAC composition (mg/L) of the North Sea oil. NPD: sum of naphthalenes, phenanthrenes and dibenzothiophenes (see table 1 in the main manuscript).

Suppl. Table S2. BLAST analysis of bacterial 16S rRNA gene sequences recovered from DGGE bands with reference in the NCBI database.

Suppl. Table S3. Sample read number, OTUs numbers, Chao1 index, Shannon index (H´), by the definition of the cutoff levels in distance units 0.03
